# Text-Book of the Eruptive and Continued Fevers

**Published:** 1892-12

**Authors:** 


*Text-book of the Eruptive and Continued Fevers.* By JOHN WILLIAM MOORE, M.D., Physician to the Meath Hospital, Dublin; Consulting Physician to the Cork Street Fever Hospital, Dublin. Pp. xxv., 535. Dublin: FANNIN & COMPANY. 1892.

If some undue delay has occurred in the publication of a notice of Dr. J. W. Moore's book, it must not be attributed to any want of appreciation of this result of his labours, for we would desire to speak of the work in terms of the highest praise. A sensible preface enjoining the necessity for the study of fever to medical students is followed by a chapter on the nature of fever, and that by two chapters on the bacteriology of what our author terms micro-parasitic diseases. To say that these last-mentioned chapters are quite up to date would be somewhat of an exaggeration, but they present a fair and careful abstract of our knowledge upon these questions a year or two ago. Of the distinctly clinical portions we can speak with unqualified approbation. The City of Dublin and Dr. Moore's appointment therein provide a happy hunting-ground for fever-study such as no other place in the kingdom could offer. Besides the other exanthemata found in this country, typhus and typhoid are treated of in full detail, the author giving, perhaps with almost undue modesty, the results of his own wide experience, and showing also his extensive acquaintance with medical literature, domestic and exotic, by copious references both in the text and in foot-notes. The treatment of typhoid fever is always a point of interest to note, as there is so much divergence of opinion among physicians as to the best manner of feeding and otherwise managing cases of this disease. In contrast to the liberal diet administered by Dr. Barr, as recorded in the notice of his book in our last issue, Dr. Moore considers one quart of milk and one pint of animal broth to be as much as is ever necessary in the way of nourishment. To antipyretic drugs he is, like most other physicians, very strongly averse, becoming "more chary than ever" in their use; and for reducing temperature, considers bathing "far in advance of all other measures." Intestinal antiseptics he does not trust to in his own practice, but notes the use of this line of treatment by many others. Limitation of space will not permit of adequate allusion to the many excellent qualities of the work. It is by far the best text-book on the eruptive and continued fevers at present before the profession, and one which every medical man ought to have by him for constant reference.

